# Bio-Absorption of Human Dentin-Derived Biomaterial in Sheep Critical-Size Iliac Defects

**DOI:** 10.3390/ma14010223

**Published:** 2021-01-05

**Authors:** Md Arafat Kabir, Masaru Murata, Mamata Shakya, Katsuhisa Yamada, Toshiyuki Akazawa

**Affiliations:** 1 Division of Oral Regenerative Medicine, School of Dentistry, Health Sciences University of Hokkaido, 1757 Kanazawa, Hokkaido 061-0293, Japan; murata@hoku-iryo-u.ac.jp (M.M.); mamata@hoku-iryo-u.ac.jp (M.S.); 2 Department of Orthopaedic Surgery, Faculty of Medicine and Graduate of Medicine, Hokkaido University, Kita 8 Jonishi 5-chome, Kita-ku, Sapporo, Hokkaido 060-0808, Japan; yka2q@yahoo.co.jp; 3 Department of Polymer and Ceramics Materials, Industrial Research Institute, Hokkaido Research Organization, Kita 19-jo Nishi 11-chome, Kita-ku, Sapporo, Hokkaido 060-0819, Japan; akazawa-toshiyuki@hro.or.jp

**Keywords:** tooth-derived scaffold, demineralized dentin matrix, bio-absorption, bone regeneration

## Abstract

The aim of this study was to evaluate the bio-absorption and bone regeneration of human tooth-derived dentin scaffold, entitled as perforated root-demineralized dentin matrix (PR-DDM), after in vivo implantation into the critical-size iliac defects. The dentin scaffolds were prepared from human vital, non-functional teeth. Thirty artificial macro-pores (Ø 1 mm) were added after removing the enamel portion. The modified teeth were supersonically demineralized in 0.34 N HNO_3_ for 30 min. The microstructure was observed by scanning electron microscope (SEM). The 3D micro-CT and histological analysis were carried out to evaluate the bio-absorption of PR-DDM at 2 and 4 months. A smooth dentin collagen surface with symmetrical macro-pores and tube-type dentinal tubules (Ø 1–2 µm) with micro-cracks were observed on the perforated region. A significant number of custom-made macro-pores disappeared, and the size of the macro-pores became significantly wider at 4 months compared with the 2 months (*p* < 0.05) evaluated by 3D micro-CT. Histological images revealed the presence of multinucleated giant cells attached to the scalloped border of the PR-DDM. The morphological changes due to bio-absorption by the cellular phagocytes were comparable to the 3D micro-CT and histological images at 2 and 4 months. Altogether, the results demonstrated that the PR-DDM block was gradually absorbed by multinucleated giant cells and regenerated bone. Human PR-DDM might serve as a unique scaffold for extraoral bone regeneration.

## 1. Introduction

Vital human teeth provide an excellent source of stem cells, matrices, trace metal ions, and multiple growth factors, which are essential for bone tissue engineering. Dentin, a major component of a human tooth, is a bone-like mineralized tissue that shares a similar chemical composition with bone (70% mineral, 20% collagen, and 10% body fluid by wt) [1]. Both demineralized dentin matrix (DDM) and demineralized bone matrix (DBM) are known to be an organic–inorganic hybrid, predominantly composed of type I collagen fibers with the remainder comprising of non-collagenous proteins (NCPs), including growth factors [1]. Insulin-like growth factor I (IGF-I), insulin-like growth factor II (IGF-II), and transforming growth factor beta (TGF-β) were identified in human demineralized bone and dentin matrix [2,3]. In addition, bone morphogenetic proteins (BMPs) derived from human dentin, which is similar but not identical to bone-derived BMPs, induced new bone formation [4,5]. Naturally, human dentin has dentinal tubules measuring 1.2–2.5 µm in diameter. Demineralization opens the dentinal tubules, increases fluid permeability, and releases BMPs efficiently [6]. Moreover, the osteoinductive performance of DDM-based scaffolds for BMPs has been successfully reported in numerous studies [7,8,9,10]. In addition, DDM has appropriate mechanical strength, is highly acid-insoluble due to cross-linking, is biodegradable, and has the ability to augment the repair process [11]. Based on these reports, human DDM can be defined as an acid-insoluble, bio-absorbable, and bone-inducing matrix that provides an appropriate environment for bone regeneration [3,12].

A bone-inducing property of rabbit-derived DDM was discovered in 1967 [13,14,15]. The animal DDM granules induced higher and faster bone formation at 4 weeks, while calcified dentin matrix (CDM) granules induced a scanty amount of bone after a latency period of 8 to 12 weeks [14,15]. In our previous studies, human DDM granules induced bone and cartilage at 4 weeks [12,16]. In addition to DDM granules, both human and animal dentin blocks in bone defects achieved successful dentin–bone union with new bone formation [17,18,19]. In addition, DDM with artificial pores has been shown to both allow cell and blood vessel infiltration through the porous structures for rapid osseointegration similar to previous study [20]. Based on the above reports, we focused on recycling the extracted tooth as a bio-absorbable scaffold with a porous structure for bone regeneration. 

Human extracted non-functional teeth are routinely discarded as infective medical waste globally. Healthy teeth could be recycled to develop bone graft biomaterials that can overcome the limitations of autogenous bone and other bony substitutes [5,21]. An ideal scaffold would be made of an absorbable material with good mechanical strength [1]. It is well-known that human dentin exhibited excellent osteoinductive and osteoconductive capabilities similar to DBM, [22,23,24,25,26]. To date, pioneering basic and clinical studies have only applied the granule-type DDM as bone graft materials [1,8,9,27,28,29,30]. Distinct from granules, as an alternative to autogenous bone block, human DDM-based block-type grafting material might play a promising role in bone regeneration [31]. In our previous publication [32], we demonstrated that the human dentin-derived block-type material entitled as a perforated root-demineralized dentin matrix (PR-DDM) contributed to active bone ingrowth in bone defects. However, to date, no study evaluated the physiological bio-absorption of the PR-DDM. Hereby, we present complimentary data specifically focused on the bio-absorption of the scaffold in a critical-size iliac defects of large animal.

## 2. Materials and Methods 

### 2.1. Perforated Root-Demineralized Dentin Matrix (PR-DDM) Preparation

**Figure 1:**
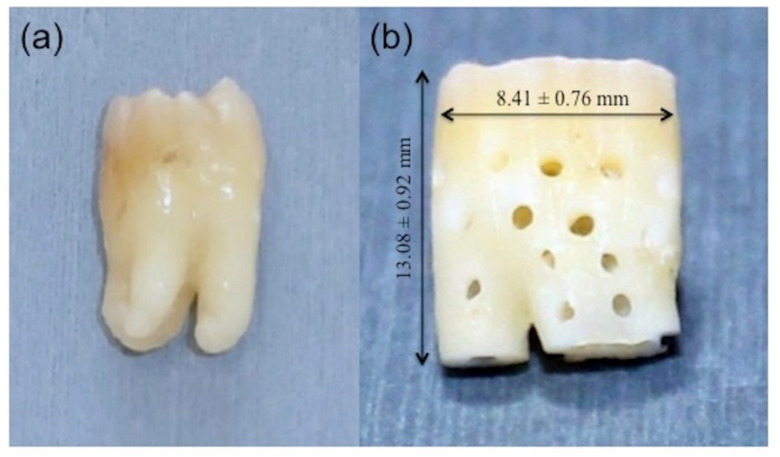
Gross images showing the transformation of (**a**) freshly extracted vital human tooth into (**b**) perforated demineralized dentin matrix (PR-DDM) (*n* = 9).

Healthy non-functional extracted teeth were used to prepare scaffolds. Twenty patients were informed of the study objectives and agreed to participate for tooth extraction by an informed consent. All patients were healthy and free of systemic diseases. The teeth were donated at the Department of Oral and Maxillofacial Surgery for the research approved by the review board of the Dental Faculty of Health Sciences University of Hokkaido. Following extractions, the teeth were stored in liquid nitrogen at −152 °C for about two weeks before surgery. After soft tissue dissection and vigorous washing with distilled water (DW), the enamel and pulps were removed with a tapered fissure bur (Mani^®^, DIA-BURS TF-11, Tochigi, Japan). A 1 mm area was cut from the apical portion using a diamond disk bur (Horico^®^, DIAMANT, Berlin, Germany). To create an optimal pore size for bone ingrowth, a small round bur (Mani^®^, DIA-BURS BR-45, Tochigi, Japan) was used to fabricate 1 mm hole, similar to in our previous study [32]. Thirty perforations were uniformly distributed to the body of the modified tooth followed by supersonic demineralization (Powersonic 603^®^, Hwashin Tech. Co., Ltd., Seoul, Korea) in 5 L of 0.34 N HNO_3_ solutions at 100 W and 60 kHz for 30 min. The liquid temperature of the sample gradually increased with an increasing supersonic time up to 30 min, but they were maintained at less than 315 K to avoid denaturing proteins, as previously reported [33]. Finally, we used an extensive rinse with DW to prepare the PR-DDM. A total of nine teeth (*n* =9) were transformed into the PR-DDM, and the average length and width were calculated as 13.08 ± 0.92 mm and 8.41 ± 0.76 mm (Figure 1).

### 2.2. Morphological Characterization

The morphological characterization of the PR-DDM using a scanning electron microscopy (SEM) (JSM-6610LA^®^, Jeol, Tokyo, Japan) was used to observe the surface texture, custom-made macro-pores, and native geometrical tunnel structures of the dentin. The scaffolds were fixed in 5% glutaraldehyde and 1% formaldehyde for 2 days. The PR-DDMs were dehydrated in a graded ethanol series (20%, 40%, 60%, 80%, and absolute ethanol) for 10–15 min and placed in vacuum desiccators for overnight drying. The following day, a total of five samples were sputter coated with 10 nm of gold and analyzed microscopically using a SEM at 1 mm and 5 μm magnifications (*n* = 5).

### 2.3. Animal Experiment

#### 2.3.1. Ethics Statement 

The animal experiment was examined by the Institutional Animal Care and Use Committee (IACUC) and was performed according to the guidelines of the Declaration of Helsinki, and approved by the Health Sciences University of Hokkaido Animal Experimentation Regulation (No: 023), and Bioscience Department of Toya Laboratory of Hokudo Co., Ltd., Hokkaido, Japan. 

#### 2.3.2. Surgical Procedures

**Figure 2:**
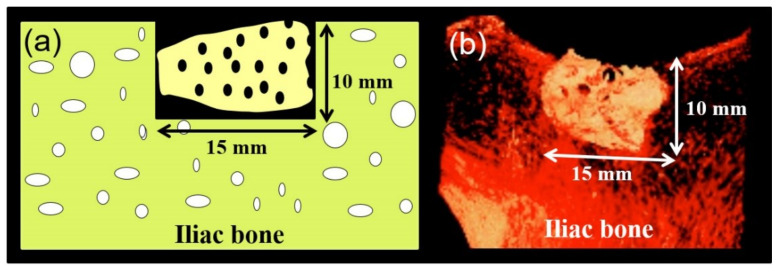
(**a**) Schematic illustration, and (**b**) 3D micro-CT images of grafted PR-DDM into critical-size sheep iliac defect.

The present study used six mature male Japanese Suffolk sheep (approximately 20–21 months old and 33–54 kg in weight). Intramuscular administration of atropine (0.06 mg/kg), xylazine hydrochloride (0.22 mg/kg), and ketamine (11 mg/kg) were used for general anesthesia. The anesthesia was maintained with endotracheal inhalation of 2% isoflurane throughout the operation. Under proper aseptic measures, a skin incision was made above the right iliac crest, and the tissues were dissected to expose the surgical site. Standardized critical-size bone defects (15 mm × 10 mm × 9 mm) in iliac crests were harvested by an oscillating saw under cooling with sterile physiologic saline, and PR-DDMs were grafted in the bone defects (Figure 2). After irrigation, absorbable sutures (Polyglactin, Vicryl^®^, Ethicon Inc., New Brunswick, NJ, USA) were used to secure the muscle flaps, and the skin incisions were closed with sterile 2-0 silk sutures. Animals were treated with intramuscular injections of penicillin G and dihydrostreptomycin (10 mg/kg) daily for 7 consecutive days. Adequate measures were taken to minimize the pain or discomfort to the experimental animals. At 2 months, three sheep and the remainder were humanely euthanized at 4 months post-operatively with intravenous injection of sodium pentobarbital-based euthanasia solution. The surgical graft sites were trimmed from the iliac crests after confirming the death of each animal and fixed in a 10% neutral phosphate-buffered formalin solution.

#### 2.3.3. Micro-Computed Tomography (Micro-CT) Analysis 

We evaluated the morphological changes due to bio-absorption and new bone regeneration of the PR-DDM at the bone defects using a micro-CT scanning device (Rigaku-mCT^®^, Rigaku, Tokyo, Japan). Samples were imaged using following parameters: 90 kV tube voltage, 160 mA of automatic tube current, 0.12 mm slice thickness, and 17 s exposure time. For three-dimensional (3D) imaging data, we used the Osirix Image processing software (version 5.6) to compare the amount of PR-DDM bio-absorption at 2 months and 4 months post-implantation. Meanwhile, the number and size of artificial macro-pores in the PR-DDM were also analyzed with a 3D reconstructive volume rendering method using the same software.

#### 2.3.4. Histological Analysis

At time intervals, the bone specimens were fixed in 10% neutral phosphate-buffered formalin solution for 1 week and decalcified with 10% formic acid for 4–6 weeks and rinsed overnight with running water. All the samples were dehydrated using ethanol (50%–100%) and processed for paraffin embedding (Vacuum Rotary, VRX-23, Mitsubishi, Tokyo, Japan). Later, a 5 µm thickness of histological sections were prepared by a microtome (Yamato Rom 380, Tokyo, Japan) and stained with hematoxylin and eosin (HE) (Wako, Osaka, Japan). The HE-stained sections were examined using an optical microscopy (Nikon Eclipse 80i, Nikon, Tokyo, Japan) for histological and histomorphometrical evaluation. 

#### 2.3.5. Statistical Analysis

The statistical values are expressed as the mean (±standard deviation, SD). Each experiment was replicated at least three times. The one-way analysis of variance (ANOVA) was used to analyze statistically significant differences between the means of two groups. The statistical significance was accepted for *p* < 0.05 using Tukey’s test. A Windows computer with SPSS software ver. 19.0 (IBM, Armonk, NY, USA) was used to perform all statistical analysis.

## 3. Results

### 3.1. Surface Topography

**Figure 3:**
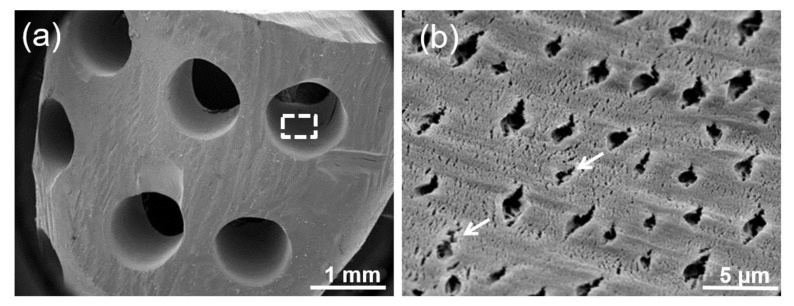
(**a**) Scanning electronic microscopy (SEM) image of PR-DDM showing smooth demineralized dentin surface with custom-made artificial macro-pores. (**b**) Higher magnification of white dotted line in Figure a showing exposed dentinal tubule with micro-cracks (white arrow) in a dense collagen matrix.

The SEM of PR-DDM exhibited a homogenous dentin surface with symmetrical artificial macro-pores (1 mm in diameter) (Figure 3a). A higher magnification image of the custom-made macro-pore region clearly revealed well-exposed tube-type pores, which are referred to as dentinal tubules, with dentinal hole diameters at approximately 1–2 μm on the dense dentinal collagen matrix surface (Figure 3b). Several micro-cracks due to the supersonic demineralization were also observed in the artificial macro-pore region (Figure 3b). 

### 3.2. Bio-Absortion Evaluation by 3D Micro-CT

**Figure 4:**
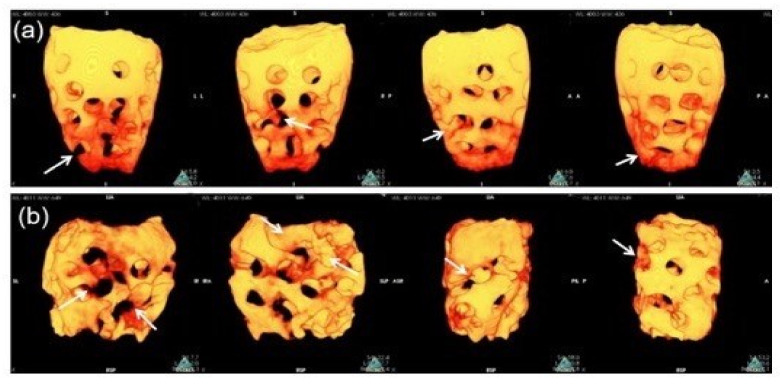
Three-dimensional-volume rendering images of PR-DDM. (**a**) Loss of custom-made perforations (white arrows) at 2 months, (**b**) Disappearance of perforations along with parts of dentinal wall (white arrows) at 4 months confirmed higher scaffold bio-absorption compared to 2 months.

We used an in vivo high-resolution 3D micro-CT to investigate and analyze the morphological changes and scaffold bio-absorption at 2 and 4 months. The difference in the PR-DDM bio-absorption was analyzed, and the images are presented in Figure 4. At 2 months, some portions of the PR-DDM were lost, especially at the lower portion; however, the entire structural shape of the material was clearly observed (Figure 4a).

**Figure 5:**
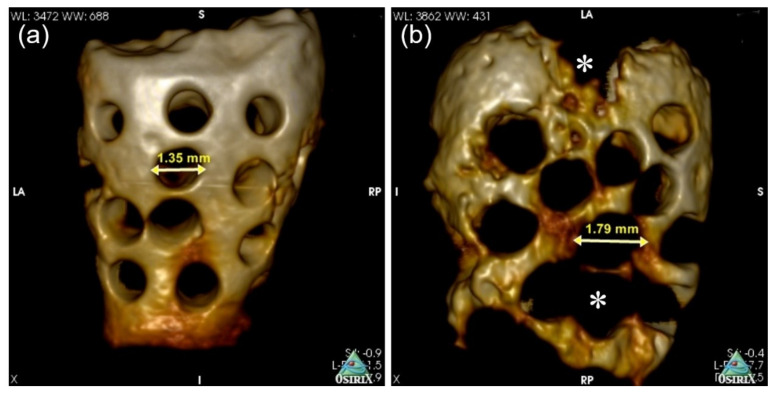
Three-dimensional micro-CT images of PR-DDM. Size of perforation (arrow) at 2 months (**a**) and 4 months (**b**). Loss of structural integrity with demineralized dentin (asterisk) due to bio-absorption at 4 months.

The structural integrity of the PR-DDM was lost especially in the middle and lower portions at 4 months due to higher levels of dentin absorption (Figure 4b). A significant number of custom-made macro-pores disappeared at 4 months compared with the initial study time and 2 months (* *p* < 0.05) (Table 1). The macro-pores became significantly wider at 4 months compared to before the graft (* *p* < 0.05), indicating advanced dentin absorption (Figure 5) (Table 1). The 3D micro-CT images highlighted the higher quantity of absorbed scaffold at 2 months compared to that at 4 months (Figure 4 and Figure 5).

### 3.3. Histological Findings

**Figure 6:**
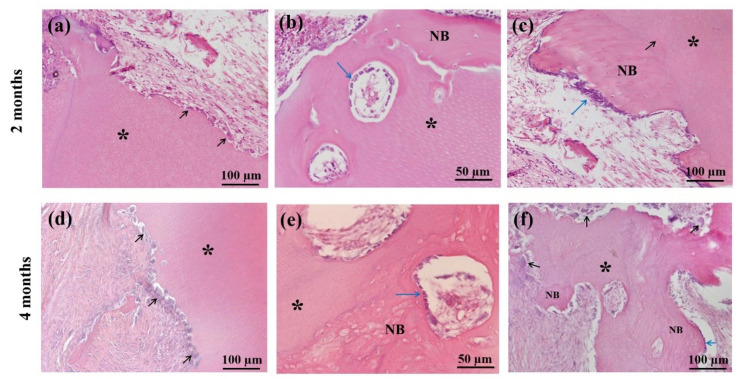
Histological evaluation of sheep iliac defects at 2 months (**a**–**c**) and 4 months (**d**–**f**) with hematoxylin and eosin (HE) staining. (**a**) Cellular phagocytosis of PR-DDM (asterisk) by multinucleated giant cells (black arrows), (**b**) New bone induction (NB) with regular osteoblast layer (arrow) by PR-DDM (asterisk), (**c**) Well-demarcated boundary (arrow) observed between PR-DDM (asterisk) and new bone (NB); note: osteoblast layer (blue arrow) on new bone, (**d**) Cellular absorption of PR-DDM (asterisk) by the higher number of multinucleated giant cells (black arrows), (**e**) New bone (NB) with mosaic-like structure induced by PR-DDM, (**f**) Simultaneous evidence of new bone formation (NB) with osteoblasts (blue arrow) and PR-DDM bio-absorption (asterisk) by multinucleated giant cells (black arrows).

Figure 6 showed histological findings of the sheep iliac defects at 2 and 4 months after the graft. The cellular absorption of the PR-DDM was observed by multinucleated giant cells at 2 months, which were attached directly with the scaffold (Figure 6a). The absorption of the PR-DDM without the presence of giant cells was also seen in several areas, and the fibrous tissue accumulation was recognized at the non-bone forming region (Figure 6a). Newly formed bone with many cuboidal osteoblasts was found directly connected with PR-DDM (Figure 6b). A well-demarcated union was observed between new bone and PR-DDM with small scaffold patches and numerous osteoblasts (Figure 6c). In contrast, light microscopy images at 4 months revealed a higher number of multinucleated giant cells attached to the scalloped border of the PR-DDM (Figure 6d). Interestingly, osteoblasts on the new bone and giant cells on the absorbed scaffold appeared simultaneously (Figure 6e). At 4 months, unorganized lamellae and irregularly placed osteocytes in the new bone matrix were observed, which were connected with PR-DDM (Figure 6f). The mean values of PR-DDM were 58.48% and 40.52% at 2 and 4 months, while the new bone occupied 4.2% at 2 months and 31.5% at 4 months, as respectively shown in our previous study [32]. These results demonstrated that the ratio of PR-DDM decreased sequentially with the increase in new bone volume. The overall findings of the histomorphometrical analysis suggested that the cellular phagocytosis was responsible for the scaffold’s bio-absorption and the new bone was entirely induced and conducted by the 3D structure of the PR-DDM. 

## 4. Discussion

The present study described the dentin scaffold’s cellular absorption and graft stability with new bone formation in critical-size defects of adult sheep. The choice of the Japanese Suffolk sheep model provides a body weight, which is comparable to adult humans. In addition, the metabolic and bone remodeling rates of these sheep are similar to humans [34]. Critical-size defects are considered to be those non-union osseous defects that will not heal spontaneously [31]. Therefore, a standardized critical bone defect was created for either 2 or 4 months to examine the morphological changes and the bio-absorption with bone regeneration of the scaffold.

Scaffold-based bone tissue engineering requires definite geometrical features for the porous scaffold. An adequate porosity of suitable size and interconnections between the pores allows higher bone ingrowth than a non-porous scaffold [35,36]. A non-porous scaffold acts as the impermeable wall for cell infiltration and vascularization [36,37]. Apart from the chemical composition, the overall geometry of the PR-DDM may also affect bone regeneration. Therefore, to prepare a better geometrical structure, to facilitate the invasion of cells and nutrients, 30 artificial macro-pores were added to the PR-DDM (Figure 1). In addition, our supersonic demineralization should improve the contact surface area of the PR-DDM (Figure 3b). Artificial perforations followed by partial demineralization might be the right design concept for recycling dentin materials as a bio-absorbable scaffold.

Many bacteria live in the human oral cavity [38]. As bacteria may contaminate human tooth-derived materials, it is important to confirm that the materials are bacteria-free before the in vivo study. Previous study confirmed that bacteria were never detected in the DDM after the supersonic demineralization [39]. Moreover, it was reported that human dentin contained a range of naturally occurring antimicrobial peptides [40]. If the PR-DDM contains the antimicrobial peptides, these peptides may play an important role in the defense against infection at the grafted site. Additionally, it is well-known that the acid treatment for bone and dentin increases their osteoinductivity and decreases their antigenicity [4]. Supersonic demineralization produced more nano- and micro-cracks, which increased the effective surface area. Thus, the body fluid will smoothly permeate through the micro-cracks in the PR-DDM [33]. These observations are scientifically important for hard tissue-derived graft material processing procedures. Therefore, we believe the advantages of the supersonic demineralization of PR-DDM should include sterilization, an increase of effective surface area, improvement osteoinductivity, and less antigenicity.

Until now, the exact mechanisms of demineralized dentin degradation in the bone tissue have not been fully clarified. Both enzymatic digestion and cellular phagocytosis are the dynamic processes involved in the organ absorption [41,42]. The supersonic demineralization of the PR-DDM leads to superficial decalcified dentin, which is exposed to the organic matrix with an inner core of mineralized dentin. The exposed collagen matrices were degraded by enzymes under different physiological and pathological conditions [42]. In vitro dentin bio-absorption by collagenase digestion was successfully evaluated by our team (data not shown). Moreover, it is well known that multinucleated giant cells degrade mineralized tissues via phagocytosis [43]. The current study has shown that partially demineralized dentin with 30 macro-pores, the PR-DDM including minerals, successfully acts as a bio-absorbable scaffold in the critical bone defect. The 3D micro-CT clearly demonstrated significant bio-absorption of the PR-DDM at 4 months compared to 2 months and the initial study time (Figure 4 and Figure 5). The loss of the structural integrity of the scaffold due to the disappearance of the artificial macro-pores confirmed the physiological absorption. The histological findings confirmed the cellular phagocytosis of the PR-DDM by the multinucleated giant cells (Figure 6a,d). Considering the aforementioned reports, we speculate that both cellular and enzymatic digestion simultaneously degraded the PR-DDM in our study. 

Biodegradable scaffolds play an increasingly important role in the bone regeneration process. It provides a bridge for new bone tissue growth into the scaffold-absorption areas and a platform for cells and growth factors to play a physiological role [44]. Our previous study extensively focused on the active bone ingrowth into the critical-size iliac defects [32]. Higher bone volume induced by the PR-DDM was observed at 4 months compared with 2 months after graft. Moreover, histomorphometric measurements also revealed a significant reduction in the PR-DDM volume with a simultaneous increase in new bone volume [32]. The current study specifically demonstrated the physiological bio-absorption of the PR-DDM essential for new bone regeneration. Gradual absorption of the PR-DDM by multinucleated giant cells and the presence of osteoblasts suggests active bone remodeling at the grafted site (Figure 6). New HE findings revealed that ankylosis between PR-DDM and new bone was successfully achieved through a combination of osteoinduction and osteoconduction. New bone in the defect site was directly connected with PR-DDM (Figure 6a–c). Bone induction in perforated spaces of the PR-DDM strongly supports the study with the perforated DBM [45]. Altogether, the results proved that PR-DDM acted as a biodegradable scaffold, and absorption of the PR-DDM provided sufficient spaces for the newly remodeled bone into the critical-size iliac defects of adult sheep. However, a lack of healthy non-functional teeth, lower concentrations of growth factors compared to DBM, and the inapplicability of the scaffold for large defects are considered limitations of PR-DDM. 

To the best of our knowledge, this is the first study that shows the bio-absorption of the PR-DDM in the critical-size iliac defects in large animals. This study highlights a new medical technology that PR-DDM might contribute as a novel scaffold for bone engineering.

## 5. Conclusions

We successfully evaluated the bio-absorption of the PR-DDM block by 3D micro-CT and bone regeneration histologically in critical size iliac defects. The fabricated perforations and supersonically 0.34 N HNO_3_ demineralization into human teeth contributed to improve the 3D dense structure and the calcified surface into a porous and irregular type. Our study indicated that the PR-DDM block was gradually absorbed by multinucleated giant cells and regenerated bone as a biological scaffold. Owing to easy fabrication in a short period of time, human PR-DDM might be recycled as an innovative biomaterial for extraoral bone engineering.

## Figures and Tables

**Table 1:** Number and size of artificial macro-pores at 2 and 4 months after graft.

Parameters	Explant Period		
Title	0 Days	2 Months	4 Months
Number of marco-pores	30	25.5 ± 3.05 *	13.3 ± 1.52 *
Size of macro-pores (mm)	1.0	1.35 ± 0.13 **	1.79 ± 0.08 **

The values are represented as mean ± standard deviation (SD) (*n* = 3) *, ** significant difference *p* < 0.05.

## Data Availability

Data sharing is not applicable to this article.
